# Making a living moment more resonant: an exploration of the role of the artist in co-creative work with people living with dementia

**DOI:** 10.12688/wellcomeopenres.19357.2

**Published:** 2024-05-30

**Authors:** Julian West, Hannah Zeilig, Timothy Cape, Lucy Payne, Clare Whistler

**Affiliations:** 1 Open Academy, Royal Academy of Music, London, England, NW1 5HT, UK; 2 London College of Fashion, University of the Arts, London, England, W1G 0BJ, UK; 3 Independent scholar, n/a, UK

**Keywords:** Dementia, Arts, Co-creativity, Wellbeing, Authenticity, Music, Dance

## Abstract

**Background:**

Despite a growth in interest in recent years in the benefits of working co-creatively with the Arts for people living with dementia, little attention has been given to understanding the role of the professional artists within this context. Our main question here is ‘How do professional artists apply their skills and knowledge in co-creative arts groups with people with dementia?’ This paper has been informed by the insights gained from a series of conversations, observations and journals that were kept by four UK based artists (two musicians and two dancers) who reflexively interrogated what they were doing during the course of an 8-week co-creative arts project with people living with dementia.

**Methods:**

The research used an empirical case study methodology, with the authors adopting a thematic approach to the analysis of the data.

**Results:**

Thematic analysis resulted in three main themes: Authenticity, Enabling Risk and Togetherness. These themes characterise the skills, techniques and specialised knowledge used by the artists during the co-creative sessions.

**Conclusions:**

Following this analysis, the article argues that the beneficial effects for people living with dementia of co-creative art-based work come about through the conscious application by the artists of their shared skills and knowledge, acquired through training and ongoing artistic practice. Rather than an assumption that ‘The Arts’ are in themselves beneficial for people living with dementia, we must consider the active role played by the artists who are so integral to the process.

## Introduction

The purpose of this paper is to contribute to a deeper understanding of the role of the professional artist within co-creative projects with people with dementia. As an approach, co-creativity involves a way of working that is non-hierarchical, where power is shared, and where the process is privileged rather than the end product ( Matarasso, 2017; Matarasso, 2019; Zeilig *et al*., 2018). Despite the sense that co-creativity is of increasing relevance to both artists and cultural institutions alike, the approach has not been widely explored, either practically or conceptually. To date, co-creativity as an approach has been investigated primarily from the point of view of those who take part and the artists’ perspective has been neglected ( Zeilig *et al.*, 2019). Here, we are concerned to examine co-creative work, from the point of view of the artists who are so integral to the process. In doing so, we aim to achieve a more integrative understanding about the way in which co-creativity works for *all* those who are involved. In ESRC and Wellcome funded projects, the authors have worked with artists who practice co-creatively with people living with dementia both in the UK and in Japan.

Our aim is to identify the artistic methods and approaches that artists adopt, the tools that are used, and to explore the role of technique. In this way, we hope to add to the nascent evidence base concerning the invaluable role of professional artists in co-creative contexts.

### Arts, dementia, and co-creativity

Dementia is a syndrome that can be caused by a number of diseases which may affect memory, cognition, and behaviour. Many people who live with dementia will eventually need help and support from others for daily activities ( World Health Organisation, 2017). In January 2023, Lecanemab was approved by the US Food and Drug Administration as a treatment for early Alzheimer's disease. However, the efficacy of this new medication remains contested and there have to date been no significant breakthroughs in prevention or cure for dementia. This is a global issue. Whilst dementia is not a normal part of the ageing process, it is known that ageing is the main risk factor in developing the condition. The United Nations *World Social Report 2023: Leaving No One Behind in an Ageing World*, states that ‘the number of persons aged 65 years or older worldwide is expected to double over the next three decades, reaching 1.6 billion in 2050, when older people will account for more than 16 per cent of the global population.’

Consequently, strategies and policies have been developed to address this significant public health issue. *The Global Action Plan on the Public Health Response to Dementia 2017–2025* ( WHO, 2017) outlines areas for action for moving towards better physical, mental, and social wellbeing and reducing the impact of the condition on people with dementia their families, carers and communities. The WHO report also notes the importance of developing person-centred and cost-effective interventions ( 2017). It is important to acknowledge that dementia manifests differently in different people and there are variations in cognitive, emotional, and physical symptoms. What has increasingly been recognised is that despite the inability to cure this condition, art projects are one way of enabling people with dementia to continue to participate in social life and have meaningful interactions ( Camic *et al.*, 2018; Fancourt & Finn, 2019; Mittner, 2022; Tischler *et al.*, 2023; Windle *et al.*, 2017; Zeilig *et al.*, 2019). Moreover, co-creative arts projects have also shown that the arts can offer new and important ways of being in the world with a dementia ( Mittner, 2022 & Lukić, 2023).

More attention therefore must be given to the ways in which people living with dementia can be cared for and included. We must also consider how public attitudes and perceptions can be challenged to address the associated stigma. Artistic practice with people living with dementia is widely appreciated as an important way of addressing stigma and enhancing wellbeing ( All Party Parliamentary Group Creative Health on Arts Health and Wellbeing, 2017; Cutler, 2015). 

### Artists and arts projects

As highlighted by Clift *et al*. (2021), the importance of artistic quality, the role of individual artists, and the ways in which they apply their skills and knowledge within the field of Arts and Health has been largely neglected in the academic literature. Similarly, there is a dearth of knowledge about the effect of co-creativity within health and wellbeing contexts upon artists’ own practice. This necessarily results in a one-dimensional understanding about such arts projects, which are only seen in terms of what they ‘do’, or their effect on those who participate, thus contributing to an instrumental view of arts projects and artists themselves. In addition, the ways in which co-creativity may enrich the lives of artists is unknown.

Nonetheless, there has been some interest in the unique contribution of professional artists to arts-based projects for people with dementia ( Coaten & Newman-Bluestein, 2013; Gilfoy & Knocker, 2009; Rose *et al.*, 2008). For instance, Coaten & Newman-Bluestein (2013) note the relevance of the dance artist as an outsider who is gradually accepted within the day centre environment. It is stressed that the dance artist may have few preconceptions about the abilities of people with dementia and was not constricted by the environment. The knowledge that artists have of existing art work, and how this can usefully inform their processes when working with people living with dementia has also been explored; for example, Jayne Lloyd (2019) describes how her knowledge of the video piece by Bruce Nauman, *Walking in an Exaggerated Manner Around the Perimeter of a Square* ( 1967–68) informed her approach to creating work which challenges perceptions of walking with people living with dementia in care home settings - a context where walking without an explicit purpose is often described as ‘wandering’, and associated with the pathology of the syndrome. Smilde *et al*. (2014) identify the use of improvisation by musicians as a way of expressing the identity and personhood of the person with dementia. This process involves the musicians working reflexively, with a high degree of self-awareness, and with an ability to be present in the moment. The benefits of working as part of a group of artists, who are able to support and inspire each other has also been noted ( Harries, 2013), especially when this group is multi-artform.

The literature shows that the professional artist can open pathways to communication, via their expertise. In addition, professionals are likely to have aesthetic standards and technical proficiency that can be positively applied when working with those who live with dementia. Thus, the evaluation of the ‘‘Finding Penelope’’ project ( Basting *et al*., 2016) reports on the importance of ensuring that rigour and high standards (in ensemble work and theatrical craft) were integrated into the process of devising and performing a play with people with dementia. The ‘Music for Life’ model is similarly based upon collaborative possibilities between professional musicians and participants ( Garrett & Crickmay, 2013); thus ‘exceptional players’ use their musical abilities to connect with even those who are living with advanced dementia. Similarly, moments of musical connection or ‘flow’ between the group members were highlighted. High aesthetic standards served to validate the whole group and raise the general level of expectation. Moreover, the benefits of having some experience of performing was noted by the artists who led the ‘Our Day Out’ project. For instance, one of the musicians recalled an occasion when he realised that he could also perform *on behalf of* the group. The work of the Artful Dementia Research Lab at UiT has importantly drawn attention to the role of relational aesthetics ( Bourriaud, 2002). The emphasis in relational aesthetics on the primacy of human relations and social context, and on art as democratic is particularly pertinent for understanding co-creative arts projects and has guided work in this area ( Lotherington, 2023; Lukić, 2023; Mittner, 2022). In relation to ‘With All’, the concept of Microtopias within relational aesthetics ( Bourriaud, 2002) sheds light on the importance of spaces that encourage dialogue and shared experience, and which foster a sense of connectedness.

As discussed earlier, co-creative practice involves working non-hierarchically with a focus upon the process. Working non-hierarchically does not imply that we elide the differences between artists and other participants – rather that the knowledge and skills of artists are used within and by the whole group to facilitate equal participation in co-creation. For artists, this has relevance because it enables an emphasis on improvisation and shared decision-making that is not always integral to their training ( Smilde, 2016). Similarly, the ability to participate fully as a member of a team is unusual for people living with a dementia and their carers. In the ‘With All’ group during a co-creative session there was no strong distinction (although there are differences) between the artists and everyone else who participated. To this extent everyone was an ‘artist’. However, in this article our specific focus has been on those members of the group who had received training as professional artists and therefore have a more practised and explicit facility with musical and dance skills and knowledge. Working co-creatively with the arts with people living with dementia has been shown to be an effective way of exploring creativity, encouraging expression and connection practically and experientially, although the field is emerging and requires further and broader research, and, we argue here, the insight from artists themselves.

## Methods

### Ethics

All of the artists involved gave written informed consent for their journals to be analysed as part of the research process, and ethical approval was granted by the Ethics Committee of University College London (approval number: 8545/002).

This article builds on the work published by the authors in two previous publications. The first of these ( Zeilig *et al*., 2018), is a conceptual piece, which outlines the concept of co-creativity, and used data from interviews conducted remotely with artists who considered their work to be co-creative. It outlines broad concepts and guiding principles for co-creativity. The second ( Zeilig *et al*., 2019) analyses data collected from people living with dementia who took part in the ‘With All’ project that took place during the Created Out of Mind residency, and comprises interviews, questionnaires, and video. The article describes the benefits for people living with dementia of working co-creatively through the arts. In this study, the intention has been to elucidate the particular role of the artists who facilitate the sessions - i.e., attempting to move towards an understanding of *how* artists work co-creatively. Although the data was generated by the artists during the ‘With All’ project, this study analyses it for the first time.

An empirical case study approach was used as a means of collating, framing, and making sense of the data. Following Yin who defined case study as a research method ( 2018) the authors adopted a qualitative, realist approach. The case study comprised 4 × 1 hour co-creative group arts sessions that took place weekly at the Hub at the Wellcome Collection in London over a four week period. The four artists were embedded units within the case. This is in line with Yin (2018: 54) in which he outlines that subunits of analysis may be incorporated within the single-case study thereby creating a more complex (embedded) design. A case study facilitates the investigation of a contemporary phenomenon (here: the role of artists in one co-creative arts project for people with dementia) within their real-world context. A case study approach relies on multiple disparate sources of evidence which all relate to the case being studied. In this instance, we have analysed reflective journals, observations, and insights from collective discussions. The musicians and dancers who participated in the *‘With All’* project took part in preparatory and reflective discussions in advance of and following each of the sessions. Drawing on these collective discussions and their own personal reflections, each kept an unstructured reflective journal throughout the project, in which they considered the ways that they were approaching working co-creatively with people living with dementia and their partners. In these journals, the artists reflect upon their individual as well as their collective practice.

### Code and theme development

The four artists each kept detailed reflective journals during the two-month project. Observational notes kept by the researchers were also included in the analysis.

The two lead authors used NVivo qualitative data analysis software to analyse and then independently code the data from these journals. An inductive approach was adopted, with the intention of identifying the ways that the artists were using their skills both consciously and reflexively during the sessions. Following an initial process of familiarisation with the data, each researcher followed a process of coding. The researchers then merged their codes, which totalled 190 in number. Through iterative coding and discussion, the two researchers collapsed the codes into code groups and finally into the three overarching themes which are discussed below. This was a process of emergent thematic analysis which is more concerned with patterns rather than frequency. In contrast to classic content analysis, this form of thematic analysis, as used by Dodds *et al*. (2008), uses empirically emergent, rather than theoretically generated themes ( Searing & Zeilig, 2017). This is a small-scale qualitative study. While our sample of five artists is limited, the data is rich and illuminative. We use it here to make some points that are relevant and interesting for scholarship on co-creativity and the role of the artist, rather than to claim any wider representative significance.

## Results

**Figure 1.**
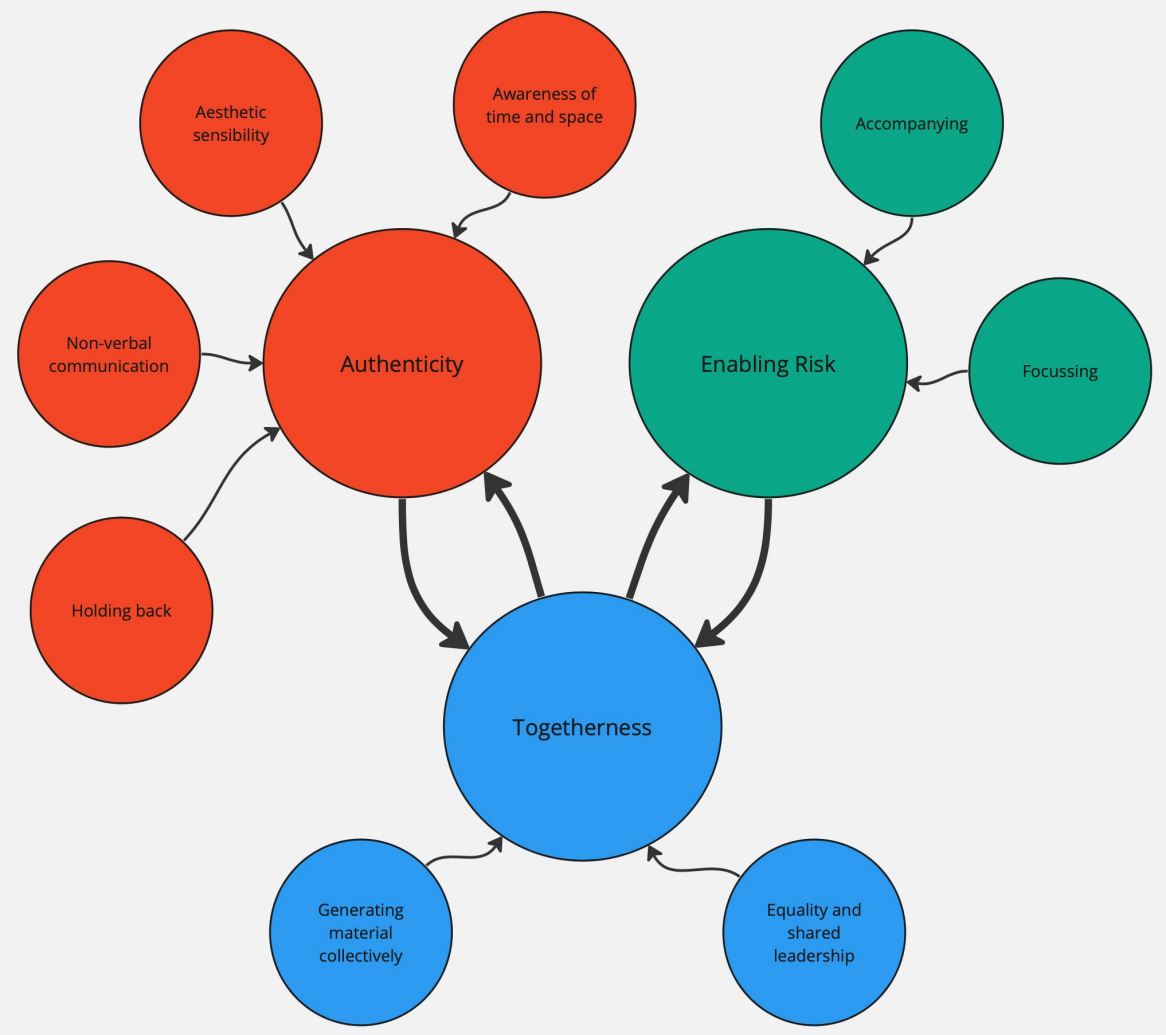
Main and subordinate themes.

The following section describes the findings from the thematic analysis. Three main themes emerged which characterise the skills and techniques used by the artists during the co-creative sessions – Authenticity, Enabling Risk and Togetherness. Each of these themes is informed by a series of subordinate themes. The relationships between the main themes and the subordinate themes are illustrated in Figure 1 below, with the main themes represented as larger circles, and the subthemes as smaller circles. As can be seen in the diagram, there is also interaction between the three main themes, and this is described later in this Results section.

### Authenticity

In their journals, the artists recorded that vital to the success of the work was the importance of working openly, honestly, and trustingly with each other and with the participants. This resonates closely with the accepted understanding of authenticity which is being true to oneself. Authenticity here, refers to the artists’ relationship with themselves and hence with the co-creative process. The act of self-reflection can be seen as a key requirement for authenticity. The artists’ reflections clarify that their willingness to explore and express their own vulnerability contributed to working authentically during the sessions, as they also explored and expressed a range of emotions alongside the other participants. One artist recorded that the sessions were for them about ‘becoming seen’, and that there was ‘no hiding place’ –

*There is no hiding place in this project and I am terrified and excited to see what I can reconnect and discover anew in myself in this area.*

Authenticity, which emerged as central to the artists’ practice, was reinforced by the co-creative sessions. There have recently been a number of scholars interested in authenticity in relation to the lives of those with dementia ( Bartlett, 2022; Hughes, 2019; Swaffer, 2014). This analysis revealed the possibility that authentic practice for artists is enhanced by collaborating with people with dementia. The techniques employed by the artist are explored below through the subordinate themes that emerged through the coding process: *holding back, non-verbal communication, awareness of time and space, aesthetic sensibility*.

***Holding back*.** The conscious technique of holding back emerged as key aspect of authenticity. The artists who participated in ‘With All’ identified ‘holding back’ or ‘waiting’ as fundamentally important, as it allowed opportunities for participants to initiate activity of their own choosing and at their own pace. Holding back also gave time for the artists to formulate genuine and authentic responses. These periods of apparent inactivity were described as ‘lulls’:

*Treasure the lulls - it's where everyone knows it's co- created and improvised.*

One of the artists likened the ‘lulls’ to his experience of surfing; when in the water, he never felt any anxiety about whether the next wave would come or not - it was just a matter of waiting. This same sense of trust was echoed by one of the dancers, who also reflected upon the possibility that holding back and waiting not only allows for the atmosphere in the room to change, but also allows the people within it to accept that change and respond to it:

*There is also the need to trust that if nothing is happening, if one stays true and waits the space may possibly transform.*

This waiting can often result in silence - something which people can find uncomfortable. However, in the ‘With All’ sessions, allowing silence was enabling, in that it afforded opportunities for consolidation and renewal. As one of the artists reflected:

*Silence is the winter that enables the spring…*
*Silence is the conversation making itself.*
*Silence is the previous words and sounds affirming themselves.*
*Silence is us descending out of the air, back into the body and back onto the chair.*

Holding back can also allow for moments that feel open-ended, uncertain, unresolved - or polysemous. This leaves space and opportunity for participants to find their own personal responses or resolutions, or indeed to allow things to remain unresolved, experiencing this ambiguity collectively.

***Non-verbal communication*.** In their reflections, the artists identified that for much of the time, they were interacting and communicating non-verbally. For example, by choosing to become silent, they were able to cede power:

*Silence is handing over the baton. Non-explicit exchanging of leadership.*

One artist even wondered whether the communication that was possible through the art form was superior to that which could occur verbally:

*Strangely it was those with whom I have spoken to less/ have less words themselves that I felt I saw more*

Non-verbal forms of communication in the pursuit of making music and dance together may prompt more eye contact and touch within a session, with the innovative use of an artists’ technique and skills being more direct, truthful, and authentic than the formulaic phrases that are more often used in exchanges with people living with dementia. Examples from the artists’ journals illustrate the depth of the communication and connection that occurred through their confidence to work as trustingly and openly with the project participants as they would with their trained colleagues:

*C wanting his history, but in the dance his hands held so tight his gaze went so deep, yes, he said, my gaze goes right into you – I see you.*
*C musically fantastic on the tambourine. Seems like he engages in his own time, in his own way, on his own terms. Was very nice not to be leading the rhythm, but following / decorating.*
*The moment where I placed the side of my face against the palm of his hand and he moulded to me, read me.*

***Awareness of time and space*.** Through their training and ongoing practice, musicians, and dancers acquire an acute awareness of time and space. Both art forms respond to the physical spaces in which they take place. Musicians take into account the acoustics of a physical space and also the positioning of players in relation to one another. Dancers are keenly aware of their position and orientation within space, and to one another. Both art forms are also ephemeral, with works existing only in the moment in which they are being practised, unfolding over a period of time. In bringing this acute awareness to the co-creative sessions, artists were authentically bringing the fullness of their practice.

This awareness of time was reflected in the artists’ journals. Artists were aware of how long pieces lasted:

*I held the drum for J who had been co- creating with C – he had been there a long time*
*Watching the salt/dust in the egg timer, its white salt like appearance taking 7 minutes, I traced the air*

There was also an awareness of things needing to be given enough time, and for the artistic processes to unfold at their own pace in order to express their full and genuine meaning and value:

*giving ourselves the time to render ourselves to that moment which widens the field.*

The idea of ‘space’ occurred often in the artists’ journals, with different meanings. The data shows an awareness of the actual physical space that the sessions took place in, and how people were positioned within it, and the effect of this upon the group. In the following quote, one of the musicians reflects upon the possible negative impact upon the integrity of the group, with a division being made by the seating arrangements:

*I also felt uncomfortable about where I was sitting. I felt the circle was a little unbalanced- there was an artists side and a participants side.*

The term ‘space’ was also used often with reference to ‘leaving space’, thereby maintaining the possibility of unexpected contributions by the participants, and the shared sense of creative exploration:

*The 'not- knowing', the shared lack of direction/the SPACE.*
*because that is where the possibilities lie... and where the shared experience begins*

***Aesthetic sensibility*.** An appreciation of beauty and sense of personal taste is part of the human condition. However, intrinsic to the training of an artist is the notion of creating and/or performing and presenting discreet works or pieces. In bringing this quality of their training and practice to the project, artists shared themselves more fully with participants. In the ‘With All’ project, there was often a sense that the co-created artistic material fell into a series of discreet pieces, each of which had its own distinct character and identity. The artists frequently referred to pieces, or even gave names or titles to episodes within the sessions, for example:

*‘Leaf dance’ was a release after (the) profound moment before.*
*The piece with singing was a lullaby to A*
*D- wit- performance wonderful first piece with words*

As a result of their training and experience, the artists brought an appreciation of the material being created by participants, seeing the beauty in it, and responding accordingly:

*C and I making grids with our hands across the ground which felt a response to C*
*objects arrayed on the ground became passageways to move through*

The artists’ knowledge of the arts sometimes gave them references that assisted this, providing them with a basis to respond that was concordant and enhancing of the participant’s improvised material:

*The ’relief’… set up K, M, J all in a row could be Egypt 3 high priests section, wooden crown on K’s head*
*R is in a John Cage world..*

For artistic material to attain a sense of being a piece, it is necessary for it to have structure. The artists noted that throughout the improvisations, they were considering and working towards this sense of structure. This included being aware that a physical movement, or sound made on an instrument by one of the participants could be interpreted and responded to as an intention to begin a new piece. As one of the artists noted:

*It was a completely instinctive reaction to what he (participant) was hearing and for me this was when the session truly began.*

Once a piece was underway, there were various ways in which the artists developed and sought to give it a coherence and integrity. These included a recognition and repetition of musical or physical gestures, motifs, that served as anchor points for the group to return to within the improvisations:

*A first small one then a return through the session*
*Later I used the tiny light, it kept coming back so a composition of the whole piece contained this return*

Artists were also aware of pieces coming to an end, or being completed, either by themselves or collectively. Acknowledging this completion was important for the sense of group identity:

*We had a moment after completing a piece where we sat there in companionable silence*

Moments such as this also support the theme of Togetherness which will be explored later in this article.

### Enabling risk: improvisation

For the artists, improvisation was central in facilitating spontaneous responses to the participants - who were also improvising. Improvisation was also noted as a way of connecting with the artists’ own personal creativity, and as a way of bringing them into relationship with others:

*What is improvisation? This is personal and there are distinctive degrees. For me it is a process of falling into a moment in time and being alive to the choices presenting themselves in that space. Normally and in my own experience this is felt amongst fellow participants.*
*Improvisation is loosening control and being a witness to yourself.*
*Listening becomes more important than making sound.*
*Leaving space becomes essential*

Improvisation as described by these artists is a method for accessing creative risk and thereby the generation of material, both individually and collectively. As has been mentioned, the importance of facilitating a context where participants felt able to take creative risks was highlighted by the artists:

*there needs to be an element of creative chaos before something of clarity can emerge from it*

The artists considered enabling a willingness to improvise, and with that a growing confidence in taking risks, experimenting, and stepping into the unknown, as fundamental to the group’s creative process:

*I was aware of how difficult it is to allow people to feel the uncertainty of not knowing what will happen next and yet I am so aware that ..that is the very essence of the starting point for co-creativity.*

In their journals, the artists explored the ways in which they drew upon their artistic skills and knowledge to support participants in engaging positively with this sense of uncertainty.

In the following sections, the subordinate themes of Focussing, and Accompanying explore techniques that were employed by the artists to facilitate improvisation and creative risk taking in the group.

***Focussing*.** At points in the *With All* sessions, the freedom of improvisation resulted in several different musical or movement ideas happening simultaneously. Mindful of the possible negative effects of this upon the participants living with dementia, and thereby the erosion of their confidence to improvise, the artists reflected upon the ways in which they mitigated against this. One technique was to consciously draw the group’s focus to one individual’s emergent creative material by supporting it with their own improvisation, creating material themselves that was sympathetic with it and thereby amplifying or reinforcing it. This had the effect of drawing the group’s attention and focus to a single artistic idea, a cohering of the group’s creative process, and a renewed sense of clarity. One of the artists wrote of the way in which they used their knowledge of musical elements to attempt to do this:

*(by employing) clarity in musical textures to help bring focus.*

In contrast to this technique of reducing the different artistic ideas happening concurrently, the artists also on occasion improvised material that brought together and included these differences, thereby creating an over-all sense of inclusion and acknowledgement. In this sense, the artists were widening the lens of the group, bringing the focus to the group as a whole, and creating a piece within which everyone’s individual ideas formed a coherent whole. Artists reflected upon the ways in which they remained aware and sensitive to all members of the group, ready to improvise, acknowledge and include any creative response:

*I am constantly trying to be intuitive, to 'read the room', the moment, to watch for any signals, any moment that someone is initiating/responding.*

Both of these approaches can be seen as methods that were employed by the artists to enable individual creative exploration and risk taking whilst maintaining a sense of psychological safety for the group.

***Accompanying*.** Accompanying was used by artists to support participants in taking creative risks, extending the groups’ creative exploration. The artists paid attention to and focussed upon the emergent material being created by the participants, while simultaneously generating material of their own which was supportive and complementary. In musical terms, this is known as accompanying, and refers to supporting the main line of the music with an additional musical part, for example a guitarist might both support and enhance the vocal line of a song. Accompaniment is not just ‘going along with’ but is active and enabling. For example, a tentative musical gesture offered by someone can be accompanied in such a way as to acknowledge it as artistic material - maybe by repeating it, harmonising it, extending it, adding pulse and rhythm or ornamenting it. As one of the artists noted:

*By supporting them you empower them to go further*

It should be noted here, that in a reciprocal fashion the artists were enabled to ‘go further’ by working in tandem and alongside people with dementia and that this reciprocity was one of the distinctive features of the co-creative process. Whilst the subordinate theme of Accompanying can be seen to be an enabler of creative risk-taking, there are also clear ways in which it contributes to the next main theme to be discussed – that of Togetherness.

### Togetherness

A sense of Togetherness, or of collective belonging, emerges from the artists’ reflections as an over-arching theme. The work described in the preceding themes of Authenticity and Enabling Risk was pursued in order to create an experience of collective belonging that included artists and project participants equally. The artists wrote in their journals about some of the techniques and approaches which they were able to employ once the sense of Togetherness had been established, and which also reinforced it. These were techniques to facilitate the collective generation of material, which is a crucial aspect of co-creativity, and actively supporting equality within the group through consciously sharing leadership with them.

***Generating material collectively*.** The artists’ used multifarious techniques for the collective generation of artistic material. It was possible to utilise these techniques because the sense of togetherness had been established, and they were also important ways of building upon it.

Waiting and allowing space for participants to initiate has already been discussed. However, as equal members of the group, at times artists followed their own impulses, sharing their own creative offerings:

*I started with a twisting turning on a chair*
*I let my feelings take me to an internal place, was there for them, but for once I was staying with my inspiration, my own journey of improvisation*

Artists also actively invited participants to share their own creative response to a stimulus in non-directive ways:

*Instruments were handed around, offerings towards a mightier contribution*
*A. at the start, three times did not want the flower, then did. The game of it, the choice of it*

Once initial material had been created, either by one of the artists or by an individual participant, there were then ways to build on this to develop the material co-creatively. This might be through the artists improvising material responsively in a turn-taking, back and forth fashion (call and response), repeating material back sequentially (echoing), copying material concurrently (mirroring), and developing musical motifs and physical gestures by adding their own additional material to that generated by participants. It also happened by adding further layers of accompaniment to the material as discussed above. Typically, other participants also engaged in some of these accompanying techniques, with artistic material being added or responded to by all members of the group, with pieces evolving in unplanned ways. As one artist noted:

*Like building a building, block by block without knowing the ﬁnal design.*

This organic, unplanned evolution of pieces was a significant way in which the shared identity of the group was enhanced.

***Equality and shared leadership*.** The sense of togetherness was dependent upon an equality within the group, with relationships between artists and other participants being parallel rather than hierarchical. As described above, the artists consciously sought to foster shared leadership within the group, and thereby a context where each person was able to exercise power as and when they chose to. This was referred to as an ebb and flow:

*I was thinking about the ebb and flow of co-creativity. I think in my mind I have thought of 'equality' being at the very heart of co-creativity. As I reﬂect more I see that there is a real power in these moments of equality but that the balance in partnership to achieve those moments is in constant ebb and ﬂow*

Also present in the data was a recognition that both the artists and the people with dementia were growing and developing together, that everyone was able to make a contribution, and that responsibility for the group was shared:

*Do I change, do they change? One needs the other*
*At one point I danced with R and she held me and rubbed my back*
*who was holding who*
*we did both*

## Discussion

The findings that have emerged from our analysis of four artists’ reflections reveal some of the ways that co-creativity works and how the artists draw on their knowledge and skills, in order to co-create. The artists were all involved in a single project with people with dementia and were able to interact in ways that were not solely cognitive. As noted elsewhere ( Hughes, 2014; Lukić, 2023; Mittner, 2022; Zeilig, 2014), artistic practice can help to understand dementia in a broader context – more feelingly. Similarly, collaborating with people with dementia can give artists new perspectives on what art is. This important reciprocity is fundamental to co-creative practice, hence the professional artist is not privileged ‘over’ other participants, rather they are enabled to work alongside ( Lukić, 2023) and with the whole group.

### Wellbeing and authenticity

As recognised by the artists in their reflections, authenticity was a central part of their practice. Recently authenticity in relation to both ageing and specifically dementia has been cogently theorised ( Hughes, 2019; Hughes *et al.*, 2022; Sabat, 2019). Similarly, the authors’ previous work identified the importance for wellbeing of acknowledging unease, discomfort or illbeing. This renders the concept of wellbeing more truthful or authentic ( Zeilig *et al*., 2019). The philosopher and psychiatrist Hughes cites Laceulle (2018) ( Hughes *et al*., 2022), who provides compelling reasons why authenticity should be used in the socio-cultural narratives that surround ageing. Authenticity is a notion which allows both an acknowledgement that there is the potential for growth in later life as well as recognition of increasing vulnerability and the nearness of death. Thus, the notion of ‘authentic ageing’ provides a richer conception of ageing than those usually discussed. Moreover, on Hughes’ and Sabat’s conception ( 2019; 2021; Sabat, 2001) if we understand ourselves as socially constituted, then we are ourselves partly because we exist alongside others. To be a self is to be embedded in a context and therefore authenticity involves being true not simply to yourself but also to others. Art is about connection with others and the artists cogently reflect on how fundamental authentic connection is and outline some of the ways in which this can be achieved. For instance, they outline that holding back and showing awareness of time and space can facilitate more authentic, creative connections. The lack of forcing activity or artificially filling a moment contributed to the sort of social and relational authenticity also explored by Hughes (2021).

### Normalisation of creative risk-taking

For artists, experimentation and creative risk is a necessary element of their development and their practice when making new work. Indeed, Vincent van Gogh, an artist revered for his creativity wrote to his friend Anton van Rappart ( Van Gogh, 1885):

*I keep on making what I can’t do yet in order to learn to be able to do it.*

Miles Davis expressed something similar in this quote ( Szwed, 2012):

*I’ll play it first and tell you what it is later.*

This study demonstrates the ways in which the professional artists were able to draw upon their own experience, technique, and higher level of comfort with experimentation, uncertainty and risk-taking in order to support the participants in exploring their own creativity together. The artists were enabling conditions which were optimised for creativity to flourish, both in terms of psychological safety and freedom as theorised by Carl Rogers (1954) and Mihaly Csikszentmihalyi (1996). In the words of community musician Lee Higgins ( 2009, 2012), the artists were able to give the group a sense of ‘safety without safety’, in which creative risk taking became normalised. For people living with dementia, opportunities to take risks of any kind may be much reduced as a result of the perceptions and concern of others who judge that any kind of risk (including emotional risk) must be avoided at all costs. This study shows that the training and experience of professional artists can give them important skills, ability and confidence to engage people living with dementia in creative risk. That this has the potential to be beneficial and empowering for people living with dementia has been shown in our previous work ( Zeilig *et al*., 2018).

### A shared artistic language

The training and the practice of the artists have given them a shared language and implicit understanding of artistic processes that enabled them to work together. Being able to communicate with one another through the tools and techniques of their art forms enabled them to work spontaneously and non-verbally. Musicians and dancers shared a common, internalised understanding of elements of their art forms, such as roles to be taken (solo / duet / accompanist), tempo (the speed of the pulse of the material), articulation (whether the material was smooth / flowing or detached / angular) and the structures of pieces being created. This shared understanding enabled them to respond in ways that were most appropriate for the material generated and offered by participants, appropriately combining elements for maximum effect. The conductor Daniel Barenboim (2008) considers this need for the careful balancing of the elements of a piece of music in order for its full meaning to be conveyed:

*In music, everything must be constantly and permanently interconnected; the act of making music is a process of the integration of all its inherent elements. Unless the correct relationship between speed and volume is established, such integration is not complete and it therefore cannot be called music in the fullest sense of the term.*

In addition, knowledge of and reference to existing works of art sometimes brought the artists together in a shared understanding of how to respond collectively to material generated by participants, so that the participant’s work was supported and enhanced. For example, Clare’s comment ‘ *R is in a John Cage world’* immediately communicated that R’s rhythmic, non-sensical repetition of words could be related to in the same way as the *Story* movement from Cage’s *Living Room Music* ( 1940). This not only served as providing a ‘way in’ for the artists, but also re-contextualised R’s words as purposeful and meaningful when they might more commonly have been seen as problematic symptoms of her dementia.

### Going further together

The artists experienced a sense of collective belonging within the project. Thus, material was generated collaboratively and the sense of equality amongst all those within the project was both established and then reinforced, leading to the ebb and flow of power and a truly shared leadership. Underlying the togetherness experienced by the artists was a profound mutuality of trust. The importance of a feeling of togetherness has been cogently theorised by Sennett (2013) in his book *Together: The rituals, pleasures and politics of cooperation.* Here he outlines the relevance of cooperation as a social asset and as a craft:

*Cooperation oils the machinery of getting things done, and sharing with others can make up for what we individually lack. Cooperation is embedded in our genes but… it needs to be developed and deepened. This is particularly true when we are dealing with people unlike ourselves* (2013:ix)

These observations have resonance for the ways in which the artists forged togetherness with a range of ‘unlike’ people, including themselves. This points to the possibilities that artistic co-creativity has for encouraging ethical collaborations, ways of working in which we embrace our own limits and extend these by cooperating with others.

## Limitations

This study is the first to consider the ways in which the specific skills and knowledge of professional artists contribute to co-creative work with people living with dementia. Although the data from the artists’ journals is rich and extensive, our findings are limited in that the study is small scale, limited to four artists who are demographically similar – all four are white British, living at the time in the Southeast of England. With All, like most arts projects, was tightly time-limited due to funding constraints – this limited the amount of data collected. The study is limited also in that it draws only upon the data from the artists’ reflective notes and journals. Moreover, the authors’ perspectives as researchers and artists within a Western aesthetic and using a traditional qualitative research paradigm imposes certain limitations on how the data were created, analysed and interpreted. Replication of the study may be challenging, in that the study worked with a particular group of artists who are experienced in co-creative practice with people living with dementia, and sharing of their methodology with others may present difficulties.

Future studies should seek to gather data from a larger and more diverse group of artists, and over a more sustained period, using a wider range of research methods.

## Conclusion

This exploratory study suggests that the knowledge and skills of professional, trained artists contribute significantly to the beneficial effects of co-creative work with people living with dementia. Although risk is not something generally encouraged in work with people living with dementia (in fact, work with this population is more often risk-averse), it is an essential aspect of co-creativity, and the artists employ and distribute their experience and skills to promote creative risk-taking. Through co-creativity, and the deliberate actions of the artists, togetherness, connection, and community are created. The benefits of co-creativity are therefore not necessarily inherent in the art form itself, but in the equality and shared creative journey. Authenticity is a key element to the success of the attempt to work co-creatively, with artists sharing their skills and artistic methods with transparency and generosity, engaging fully in the creative process themselves as equals.

## Data Availability

Figshare: With All Artists Journals.pdf https://doi.org/10.6084/m9.figshare.22759322.v1 ( West, 2023) This project contains the following underlying data: With All Artists Journals.pdf Data are available under the terms of the Creative Commons Zero “No rights reserved” data waiver Attribution 4.0 International (CC BY 4.0).
