# Supplementary material for: Making a living moment more resonant: an exploration of the role of the artist in co-creative work with people living with dementia
Source: Wellcome Open Res. 2024 Jul 24;8:580. Originally published 2023 Dec 28. [Version 3] doi: 10.12688/wellcomeopenres.19357.3 (PMC11109536; doi:10.12688/wellcomeopenres.19357.3)
Supplement: Peer review history

# Reviewer report: Reviewer response for version 3

**DOI:** 10.21956/wellcomeopenres.25165.r90724

**Published:** 2024-08-15

**Authors:** Andrew Newman

**Affiliations:** 1 Newcastle University, Newcastle upon Tyne, England, UK

This should be changed 'As noted in the World Health Organisation (2017)’ – should be 'as noted by the World Health Organisation (2017)’.

 The below is a misunderstanding of methods – of course the study could not be replicated, no one would expect it to be.

 'Replication of the study may be challenging, in that the study worked with a particular group of artists who are experienced in co-creative practice with people living with dementia, and sharing of their methodology with others may present difficulties’.

Is the work clearly and accurately presented and does it cite the current literature?

Yes

If applicable, is the statistical analysis and its interpretation appropriate?

Not applicable

Are all the source data underlying the results available to ensure full reproducibility?

Yes

Is the study design appropriate and is the work technically sound?

Partly

Are the conclusions drawn adequately supported by the results?

Partly

Are sufficient details of methods and analysis provided to allow replication by others?

Yes

Reviewer Expertise:

I’m professor of Cultural Gerontology in Newcastle University – I’ve undertaken work on arts activities for people living with dementia.

I confirm that I have read this submission and believe that I have an appropriate level of expertise to confirm that it is of an acceptable scientific standard.

# Reviewer report: Reviewer response for version 3

**DOI:** 10.21956/wellcomeopenres.25165.r90725

**Published:** 2024-08-08

**Authors:** Robyn Dowlen

**Affiliations:** 1 The University of Manchester, Manchester, England, UK

Thank you for taking the time to address the comments made on the previous version of this article. I think this is a very clear and well articulated article which highlights the important role of artists in co-creating work with people living with dementia.

Is the work clearly and accurately presented and does it cite the current literature?

Yes

If applicable, is the statistical analysis and its interpretation appropriate?

Not applicable

Are all the source data underlying the results available to ensure full reproducibility?

No source data required

Is the study design appropriate and is the work technically sound?

Partly

Are the conclusions drawn adequately supported by the results?

Yes

Are sufficient details of methods and analysis provided to allow replication by others?

Partly

Reviewer Expertise:

Music and dementia, specifically 'in the moment' and embodied approaches

I confirm that I have read this submission and believe that I have an appropriate level of expertise to confirm that it is of an acceptable scientific standard.

# Reviewer report: Reviewer response for version 3

**DOI:** 10.21956/wellcomeopenres.25165.r90726

**Published:** 2024-08-07

**Authors:** Elizabeth Barry

**Affiliations:** 1 University of Warwick, Coventry, England, UK

No further comments.

Is the work clearly and accurately presented and does it cite the current literature?

Yes

If applicable, is the statistical analysis and its interpretation appropriate?

Not applicable

Are all the source data underlying the results available to ensure full reproducibility?

No source data required

Is the study design appropriate and is the work technically sound?

Yes

Are the conclusions drawn adequately supported by the results?

Yes

Are sufficient details of methods and analysis provided to allow replication by others?

Yes

Reviewer Expertise:

Literary studies, medical humanities, age studies.

I confirm that I have read this submission and believe that I have an appropriate level of expertise to confirm that it is of an acceptable scientific standard.

# Reviewer report: Reviewer response for version 3

**DOI:** 10.21956/wellcomeopenres.25165.r90722

**Published:** 2024-08-06

**Authors:** Richard Ward

**Affiliations:** 1 University of Stirling, Stirling, Scotland, UK

No further comments / suggestions.

Is the work clearly and accurately presented and does it cite the current literature?

Yes

If applicable, is the statistical analysis and its interpretation appropriate?

Not applicable

Are all the source data underlying the results available to ensure full reproducibility?

Yes

Is the study design appropriate and is the work technically sound?

Yes

Are the conclusions drawn adequately supported by the results?

Yes

Are sufficient details of methods and analysis provided to allow replication by others?

Yes

Reviewer Expertise:

Dementia Studies

I confirm that I have read this submission and believe that I have an appropriate level of expertise to confirm that it is of an acceptable scientific standard.

# Reviewer report: Reviewer response for version 1

**DOI:** 10.21956/wellcomeopenres.21442.r72925

**Published:** 2024-06-20

**Authors:** Richard Ward

**Affiliations:** 1 University of Stirling, Stirling, Scotland, UK

Many thanks for the opportunity to review this paper. It is well written, reflective and makes a useful contribution to the field of dementia studies. The paper has a clear structure, further supported with the addition of the thematic map/diagram and careful use of headings and subheadings. I have a handful of thoughts and suggestions to share with the authors and one or two questions to pose.

1. You make the point that the role and perspectives of artists themselves has often been overlooked in the dementia studies literature and this provides the rationale for your paper but I wonder if it is worth reflecting on why this gap exists and the implications of it, not least in terms of how the arts are framed within dementia care and research.
2. In the opening paragraph you talk of co-creative projects as characterised by a non-hierarchical and power-sharing approach and this point is echoed a number of times throughout the paper. The impression given, is that these are prerequisites to co-creative working, almost as a starting point rather than something that co-creative endeavours are working towards or aspiring to. I would encourage you to reflect a little further on the assumptions that underpin these assertions. Your data certainly supports the impression that the artists are offering a degree of control to the participants in terms of how the sessions progress and the direction they take but I’m not convinced the evidence supports your claim that the balance of power is being shifted between artist and participant or that we can even assume that the artists are in a position to relinquish power even they wished to. And, when you talk of the non-hierarchical nature of the encounters, my question is how this is judged and from whose perspective. You’ve made a case within the paper for the specific skills and understanding that the artists bring to the encounters as facilitators and as a catalyst for action, so what are the grounds for arguing this is a non-hierarchical encounter given this degree of expertise?
3. The paper does well to both explain and flesh out notions of authenticity and how the data illuminates our understanding but perhaps slightly less so in respect to ideas of risk, which is a hugely loaded term for dementia care and carries specific meanings and logic associated with notions of harm. It seems to me that you’re talking about something very different when you refer to risk in this paper and perhaps it would be worth expanding on that a little given the likelihood of a practitioner audience for this paper?
4. I was really struck by the quotes that you shared from the artists themselves, in part because their language an interpretation is very different from the kind of reflective discourse used in areas such as nursing or social work. Collectively the data make an important point about the unique contribution of the arts to our understanding of relations with people living with dementia to an extent that I wonder if there is scope to reflect on this toward the end of the paper. These encounters appear to be opening up new knowledge but also new ways of knowing that have direct implications for care and support.
5. On this question of the data, you have used them largely in an illustrative fashion to support a series of well made points but are there grounds to focus a little more on how things are said by the artist as well as what they re saying? There were a number of succinct and very insightful points being made that stood out for me as I read the paper, for instance one of your artists reflects ‘silence is the previous words and sounds affirming themselves’ which captured something significant about pace and engagement in a dementia context.
6. I really appreciated your emphasis on process (distinct from the more outcome driven framing that so much healthcare research leans toward for interpreting arts-based encounters). I wonder if there’s an undercurrent here that might be pulled out a little further in the discussion? Throughout your analysis you are touching not only on the ephemeral and emergent nature of these encounters but also the value of accommodating uncertainty, possibility and even chaos which resonates with wider debates in ethnographic research (Akama Y. et al, 2020 [Ref 1]) which speak to the value of a relational ontology.
7. And building on this point, I also would have liked to see a bit more reflection in the discussion on what outlasts the encounters being described. Not least in terms of creating a sense of collectivity and belonging (but also perhaps in helping people feel more comfortable in the face of uncertainty) that goes beyond the usual research focus on measurements of wellbeing or quality of life and efforts to specify how long such effects continue after the event.
8. Finally, and in respect to the three main themes identified in the paper, is there something to say about their connection and interaction? Is authenticity a prerequisite for enablement of risk? Is a sense of togetherness a product of collective risk-taking?

I hope at least some of the above points are useful and thanks again for the chance to review this lovely paper.

Is the work clearly and accurately presented and does it cite the current literature?

Yes

If applicable, is the statistical analysis and its interpretation appropriate?

Not applicable

Are all the source data underlying the results available to ensure full reproducibility?

Yes

Is the study design appropriate and is the work technically sound?

Yes

Are the conclusions drawn adequately supported by the results?

Yes

Are sufficient details of methods and analysis provided to allow replication by others?

Yes

Reviewer Expertise:

Dementia studies

I confirm that I have read this submission and believe that I have an appropriate level of expertise to confirm that it is of an acceptable scientific standard.

## References

[1] : Uncertainty and Possibility.2020; 10.4324/9781003087274 10.4324/9781003087274

# Response

**Published:** 2024-07-18

**Authors:** Julian West

**Affiliations:** Open Academy, Royal Academy of Music, London, England, UK

Thank you so much for your engagement with this article, and your comments and questions have raised many fascinating lines of enquiry which we would like to pursue. For example, it would be interesting to reflect more deeply on why the role and perspective of artists have been overlooked; we suspect this is intimately connected with how the arts are framed in dementia care, as an ‘intervention’ which can lead to specific outcomes. There has therefore been much less focus upon the craft of the artist. However, this does need much more careful and considered thought.

 As the lead author of this article and practising musician working in this field and have noted the difficulties in finding shared language with researchers. This may be another contributing factor as to why the perspectives of artists are often overlooked.

 We agree that it would be very interesting to reflect further upon what a non-hierarchical and power-sharing approach means in the context of co-creative work. This is something that would like to explore more deeply in other work. However, we do think that the **intention** to work non-hierarchically is a prerequisite to co-creative working, even though this is difficult to achieve in practice. It is true that artists are not always in the position to relinquish power, even when they wish to, but even the desire to share power is important here – you may be interested in concepts of nutrient power which are explored by Rollo May in his book ‘Power and Innocence’ (1998).

 Thank you for highlighting the topic of risk, and we agree that this is a loaded term in relation to dementia care. When working on the With All project, there were several occasions when we had to think really carefully about how to negotiate risk. On reflection, we do think in the article that we make it clear that we are discussing creative risk, but there is more to explore in thinking about how this interacts with both emotional and physical safety.

 Your point about the connection and interaction of the three main themes was also helpfully noted by other reviewers – we have addressed this in the revised version of this article.

 With regard to your other points, we agree that these are all incredibly important, but feel that they lie beyond the focus of this article. We hope to explore them further in future work.

# Reviewer report: Reviewer response for version 1

**DOI:** 10.21956/wellcomeopenres.21442.r72928

**Published:** 2024-06-20

**Authors:** Elizabeth Barry

**Affiliations:** 1 University of Warwick, Coventry, England, UK

This is a very rich, important and distinctive article about the artists' role in, and experience of, co-creation in applied arts work involving people living with dementia. It is original and valuable in considering the experience of artists in the co-creation process of such work, identifying what their abilities, experience, skills and techniques offer to the work. It is also notable in considering the role of aesthetic factors and even aesthetic value in such work--a revealing aspect of the current study that bears on the authenticity of the work and people's relationship to it, and the importance of doing something meaningful together. A high level of skill on the part of the artists and musicians involved also facilitated the use of improvisation and 'safe' creative risk-taking within a carefully and skillfully maintained artistic whole with a beginning and end. The article gives a nuanced exploration of these factors in the co-creation of activities with genuinely shared leadership and collective decision-making, which thereby fostered togetherness and connection. 

 The methodology was considered, appropriate and well-explained, and the work built on previous activity and research by those in the group, as well as the most pertinent scholarship in applied arts and dementia studies. The use of emergent thematic analysis allowed for the most significant themes to come to light in a reflective and authentic manner--as befitted the role that these principles had in the design and conduct of the project. 

 My only qualification to a warm support for the indexing of this piece is to suggest that the authors consider including a reflection of the role of aesthetic standards and factors in the conclusion as a distinctive aspect of this research.

Is the work clearly and accurately presented and does it cite the current literature?

Yes

If applicable, is the statistical analysis and its interpretation appropriate?

Not applicable

Are all the source data underlying the results available to ensure full reproducibility?

No source data required

Is the study design appropriate and is the work technically sound?

Yes

Are the conclusions drawn adequately supported by the results?

Yes

Are sufficient details of methods and analysis provided to allow replication by others?

Yes

Reviewer Expertise:

literary studies, age studies, cultural gerontology, medical humanities

I confirm that I have read this submission and believe that I have an appropriate level of expertise to confirm that it is of an acceptable scientific standard.

# Reviewer report: Reviewer response for version 1

**DOI:** 10.21956/wellcomeopenres.21442.r72924

**Published:** 2024-05-13

**Authors:** Andrew Newman

**Affiliations:** 1 Newcastle University, Newcastle upon Tyne, England, UK

This is an interesting paper that I’ve enjoyed reading. The topic is not one that has been effectively addressed elsewhere – professional artists tend to struggle to articulate their roles in ways that are easily understandable by non-artists.

 On page 3 you say Lecanemab’s efficacy is contested – is this the case? A reference is needed. There are concerns about its safety, but that’s not efficacy. See [Ref-1]

 The literature review is useful – covering the relevant material effectively.

 Is a traditional thematic approach going to work when dealing with the work of a professional artist?  What epistemology have you adopted and why? I suggest you start with this and then work forward to the method. Do some wider reading on non-positivist research methods. A scientific approach is not always appropriate, maybe an observational or ethnographic method might capture the work of a professional artist more effectively. What version of NVivo was used?

 Most of the themes identified are not unique to arts co-creation – what does a professional artist contribute that others can’t?  For example, togetherness could be achieved through any sort of collective activity. The subordinate themes perhaps work better than the main ones, which are too general. It might be that bringing these themes together is what is important rather than them individually, I’m not sure. I think a deeper engagement with creative practice (theory and practice) might be helpful. Perhaps authenticity, which is very different to the other themes, captures the topic most effectively.

 When you quote a respondent it’s worth giving some context – without identifying them. For example, an experienced artist might say something different to an inexperienced one. This allows the reader to make a judgment about what has been said.

 With qualitative non-positivist studies sample size is unimportant, a sample of one can be appropriate. You wouldn’t get a more ‘reliable’ result with a bigger dataset. Also, these studies don’t have to be replicated – it’s not relevant. If you were scientifically testing an intervention – then yes replication might be important, but you are not, you are exploring a topic. These sorts of studies are not of lesser value than a scientific one, they have a lot to offer.

 Generally, the paper is well written – however, there are some unsupported statements that need to be addressed. Page 1 ‘Dementia is a syndrome that can be caused by a number of diseases’ is taken from the WHO report directly – you need to reference it. I've not checked the referencing in the rest of the paper– please take care with your referencing.

 Have a look at:[Ref:2,3]

Is the work clearly and accurately presented and does it cite the current literature?

Yes

If applicable, is the statistical analysis and its interpretation appropriate?

Not applicable

Are all the source data underlying the results available to ensure full reproducibility?

Yes

Is the study design appropriate and is the work technically sound?

Partly

Are the conclusions drawn adequately supported by the results?

Partly

Are sufficient details of methods and analysis provided to allow replication by others?

Yes

Reviewer Expertise:

Cultural Gerontology - arts and dementia

I confirm that I have read this submission and believe that I have an appropriate level of expertise to confirm that it is of an acceptable scientific standard, however I have significant reservations, as outlined above.

## References

[1] Reference source

[2] : "Sample size for qualitative research", Qualitative Market Research.2016;19(426-432) :

[3] : Advanced qualitative research, a guide to using theory, London.2015;

# Response

**Published:** 2024-07-18

**Authors:** Julian West

**Affiliations:** Open Academy, Royal Academy of Music, London, England, UK

Thank you for clarifying the distinction between the efficacy of Lecanemab and the concerns around its safety. The Nature article does indicate some efficacy over the first 18 months of treatment, but safety concerns are persistent, and there is uncertainty concerning whether Lecanemab’s effect will endure over time. We have addressed this in a revised version of the article.

 Thank you for the really interesting query around whether a traditional thematic analysis is appropriate and agree that it would be difficult to capture the artistic processes through this method. However, we were analysing the artists’ reflective journals, and feel that the thematic analysis has revealed interesting findings.

 We agree that a non-positivist approach will be interesting for deepening understanding of the artistic processes and emotional / relational aspects of this work, and this is something we are keen to address in future work.

 We agree that there needed to be a better relationship between the main themes. This was also pointed out by other reviewers and has been addressed in the revisions of the article.

 We have amended the article to give more information about the artists and their level of experience. We have also addressed your concern about the unsubstantiated comments – thank you for bringing this to our attention.

# Reviewer report: Reviewer response for version 1

**DOI:** 10.21956/wellcomeopenres.21442.r72930

**Published:** 2024-03-06

**Authors:** Robyn Dowlen

**Affiliations:** 1 The University of Manchester, Manchester, England, UK

This is an important piece of research in the area of co-creative work with people living with dementia. There is a dearth of research in this complex area of practice and this article illuminates important role and experiences of the artists who are central to the successful delivery of co-creative work. The themes really resonated with my experiences working with artists in the field of co-creative work with people living with dementia, and I am excited for where this work may go in the future.

 Areas that could be developed further:

**Introduction**

1. The authors note in then ‘Arts, dementia, and creativity section’ that ‘…arts projects are one way of providing meaningful ways for people living with dementia to continue to participate and engage’ – but it is not clear what they are continuing to participate and engage in
2. The second paragraph of ‘Arts, dementia and co-creativity’ does not follow on logically from the points made at the end of the second paragraph and this information could be included with information about the condition earlier in this section.
3. I think there could be a greater emphasis made on how you are differentiating from the ‘professional artist’ and the ‘artist who happens to be living with dementia’ – this seems to be really important in the context of co-creativity where everyone is working in a non-hierarchical way. While I think it is really important to focus on the role of the professional artist working on this context, some consideration should be given to how people with dementia are artists in their own right, in line with creative citizenship approaches which cross-over with your theoretical development of co-creative approaches.  Consideration of this could be made in the Arts, dementia and co-creativity section

**Methods**

1. There could be more clarity in the case study approach, and the units within the case should be defined. Is it a case study of the With All project, with the artists as embedded units within the case; or is each artist an individual case study (multiple-case analysis)
2. It would also be useful to have a breakdown of the data, as it is unclear how much data you are working with. E.g. how many diary entries, lengths of entries, whether certain artists made more entries than others, the period over which diary entries were written (no. of weeks/days etc), whether all entries were written or whether artists made use of diagrammatic drawings, musical notations etc.

**Analysis **

1. While there is sufficient detail on the thematic analysis process, there needs to be more information in the methods about what the units of analysis were within the case study (Which is essential from a Yinian perspective) -  illuminate whether it was a case study of the programme (with artists embedded as units within the overarching project) or artists acting as individual case studies (multiple case-study analysis)
2. Given the emphasis placed on reflexivity of artists, it would be useful to have a reflexive statement from the authors to showcase how their interpretation of the data may have been guided by their professional backgrounds, research interests, etc.

**Results**

1. I think the definition of improvisation used by artists could come earlier in the enabling risk section to allow the reader to understand what is meant by improvisation in this context.

Is the work clearly and accurately presented and does it cite the current literature?

Yes

If applicable, is the statistical analysis and its interpretation appropriate?

Not applicable

Are all the source data underlying the results available to ensure full reproducibility?

No source data required

Is the study design appropriate and is the work technically sound?

Partly

Are the conclusions drawn adequately supported by the results?

Yes

Are sufficient details of methods and analysis provided to allow replication by others?

Partly

Reviewer Expertise:

Music, creativity and dementia, with specific knowledge on in the moment experiences held by people with dementia.

I confirm that I have read this submission and believe that I have an appropriate level of expertise to confirm that it is of an acceptable scientific standard, however I have significant reservations, as outlined above.

# Response

**Published:** 2024-04-11

**Authors:** Julian West

**Affiliations:** Open Academy, Royal Academy of Music, London, England, UK

Thank you very much for your comments and the important points made in your review. We have found them incredibly useful in revising the article in the following ways: **Introduction** **1. **This lack of clarity was also noted by Lilli Mittner,  and we have altered the sentence (please see above). **2.** Thank you for this comment. We agree and have placed this information earlier in the section. **3. **Thank you for this important point – we agree that it is tricky and yet relevant to distinguish between the ‘professional artist’ and the ‘artist living with dementia’ and indeed this was also commented on by Lilli Mittner.  We have included discussion on how we might consider the professional artist as they make use of their skills and knowledge alongside other participants within the co-creative group and how therefore together the whole group can co-create. **Methods** **1. **This is needed and we have subsequently added the following to the methods section: ‘The case study comprised 4 × 1 h co-creative group arts sessions that took place weekly at the Hub at the Wellcome Collection over a 4-week period. The 4 artists were embedded units within the case. This is in line with Yin (2018: 54) in which he outlines that subunits of analysis may be incorporated within the single-case study thereby creating a more complex (embedded) design.’ **2. **Due to word count limitations, we have not included this information in the main text. However, the diary entries are all dated and available to view – please see data availability section (a link at the end of the paper). We hope this sufficiently answers this query. **Analysis** **1. **Thank you for pointing this out. We have now clarified this is a single case study and that the artists are the embedded units of analysis (please see above). **2. **Due to word count we have not included a full statement, but in the limitations section we have added further reflections on how the authors were all approaching the study from a Western aesthetic and using qualitative paradigms that affect the overall interpretation. **Results** **1. **This definition of improvisation has now been moved to the beginning of the Enabling Risk section.

# Reviewer report: Reviewer response for version 1

**DOI:** 10.21956/wellcomeopenres.21442.r72926

**Published:** 2024-02-21

**Authors:** Lilli Mittner

**Affiliations:** 1 The Arctic University of Norway, Tromsø, Norway

The paper examines the concept of co-creativity from the perspective of professional artists working co-creatively with people living with dementia. It builds on previous studies of the same material in which perspectives of those living with dementia have been explored as well as the concept of co-creativity itself.

 The authors analyze their empirical material (reflective journals, observations, collective discussions) by means of a thematic analysis which aims to identify specific artistic methods and approaches in order to highlight the role of professional artists in co-creative settings.

 Co-creative practices with shared decision-making, non-hierarchical working practices and focus on the process is unusual for both people living with dementia and their carers as well as for many artists who are trained in the current higher arts education system. Thus, investigating co-creative practices may be beneficial for both people living with dementia, artists as well as society as a whole. If co-creative work is needed to tackle societal challenges, what does this mean for the future training of artists?

 The question of what the artists bring into co-creative work is underinvested and needs to be made explicit in interdisciplinary research. Thus, this study is of great value for future research projects in the field of art and dementia as well as other research projects that apply the arts as a method for study design, data collection, analysis, and communication of research findings.

 Shifting the research focus from the question ‘what is art’ to ‘what does the artist’ seems to me an interesting move that opens up for new knowledge that the arts and humanities bring into interdisciplinary research.

 The paper is well-written, and I have only some minor comments. In addition, I would also share three questions that are meant to inspire further thinking.

**Can everyone become an artist?**

 I understand the point of privileging the training and competencies, skills and knowledge of professional artists. Still, I wonder, if everyone can become an artist in co-creative settings? And if so, probably we need to shift the focus from people to knowledge: it is not the art itself, nor the artist and what he/she is doing, but rather the specific knowledge/skills/way of being in the world/way of seeing/approaching things and people that is of specific research interest. E.g. if there is a person living with dementia with an artistic education in the group, is this person be seen as an artist or as a person with dementia? And what would it mean for research design if one person could have different roles during the research process? If roles are not fixed, how can the specific knowledge/skill you are investigating, be captured in the data?

 It seems to me that you are arguing the artists are enhancing/making sense of chaos that appears very often in creative and liminal spaces. However, I wonder how this relates to co-creativity. If the specific training/skill/aesthetic of the artists decides (collectively) when a piece emerges and comes to an end, how does this relate to non-hierarchical practices? I think relational aesthetics (Bourriaud, 2002) can be one key to solving this dilemma. The specific aesthetic (Western, linear, rational?), that the professional artists are bringing into this study, needs to be somehow addressed in the discussion.

**How does authenticity connect to the master narrative?**

 The discussion of authenticity is not fully convincing to me and seems to lead back to an understanding of something real, realistic, positivistic, and true, which I find highly difficult in relational aesthetics. If there is someone who decides what is a piece, how to go along with it when it comes to an end and what is authentic, how does this connect to non-hierarchical practices and shared decision-making? Is authenticity something in the ‘thing itself’? I guess you mean that it emerges in intra-action, but how? And what is authenticity when we work beyond the rational mind?

**What is it that people are engaging with?**

 “What has increasingly been recognized is that despite the inability to cure this condition, art projects are one way of providing meaningful ways for people living with dementia to continue to participate and engage.» 

 Here you could problematize what it is people living with dementia are engaging with, without feeling the need to reach up to a norm. One major implication of the *With All* project seems to me to move with co-creative arts beyond care and inclusion paradigm and towards re-shaping different ways of being in the world, both for those living with and without dementia. Recent contributions in the field of critical dementia studies might be of interest to the authors (Lotherington, 2023; Ward & Sandberg, 2023). In addition, you could refer to the work by Anne Bogart (2021): The Art of Resonance. Though coming from applied theatre she outlines some interesting principles that might be similar to what you find in the study and that are significant for what ‘the artist brings in’.

**Minor comments**

**1.** **Abstract**

 You could consider adding a more specific research question. By now it is not clear from reading the abstract only, what it is that the results (three themes) are answering.

**2. Introduction & Artists and arts projects**

 There are some insights in the area that have been overseen by the authors, e.g. when addressing moments of musical connection. Here the authors could draw on the ‘Resonance’ project (Mittner, 2018) and other co-creative work conducted in Artful Dementia Research Lab at UiT The Arctic University of Norway.

 Have a look at quotation marks. I suggest using them for projects only (‘Finding Penelop’, ‘Our Day Out’, ‘The Music for Life’), but not for concepts such as flow.

**2. Method**

 You refer to previous papers that report the study in detail. Still, you could give a brief overview of the music & dance session, so the reader can gain a better understanding of where the data derives from.

 Have all authors been involved in data collection?  If so, this point could be strengthened since it adds an important layer to how data is collected and processed and goes far beyond the qualitative realist approach. You could consider drawing on the performative paradigm (Østern et al., 2021) or situated art intervention research (Mittner & Gürgens, 2021) that moves beyond the qualitative paradigm and privileges the knowledge creation through co-creative art-making as an integral part of the research apparatus.

**3. Results**

 The themes are highly interconnected and I wonder if this could be made visible as a network instead of a linear visualization of sub categories. Since you inserted cross-references already in the analysis, those could be made visible in figure 1.

**4. Discussion**

 This study would benefit from a note about from which specific aesthetic framework the artists are operating/speaking.

 In your material the artists reflect on their role to ‘support participants in taking creative risks’ and ask ‘Do I change, do they change?’ -> This needs to be reflected from a non-hierarchical standpoint.

 Who needs support, why, and how? If the co-creative sessions are equally valuable for artists as they are for people living with dementia and their careers, this should be addressed in the discussion. The quote ‘by supporting them you empower them’ seems to me symptomatic for a thinking practice that creates a hierarchical divide between we (who are trained/skilled)n and them (who are in need of care and help) rather prohibits/be in opposition to the whole idea of co-creativity as a non-hierarchical practice. The danger of privileging professional artists as art makers in co-creative practices needs to be addressed in the discussion. The whole idea of differentiating into artists and participants seems to me problematic in co-creative art practices.

 The reciprocity in co-creative practices and the impact on artists could be brought to the forefront in the discussion. «This study shows that the training and experience of professional artists can give them important skills, ability, and confidence to engage people living with dementia in creative risk. «  

 ->  Here I would add ‘and the other way around’.

 A shared artistic language seems to be crucial to me when artists enter co-creative practices as a group; to facilitate the ‘piece’ making they need to share a specific aesthetic and understanding; how this process works, could be shown in the empirical material: who do artists collaborate and tune into each other to be able to co-create beyond the rational mind and with people living with dementia? What is the difference between one 1 artist working co-creatively or being 2-4 artists? And what is the role of research artists, which means researchers who become artists in co-creative artistic and arts-based research projects?

 «This sense of togetherness importantly brought coherence to the acts of co-creation and simultaneously was an essential means of facilitating the co-creation. «

 ->  For whom is coherence important and why?

**5. Conclusion**

 I would not consider the study as a small study, but rather as an explorative study that is in its method highly novel and complex.

 All in all, I highly enjoyed reading this paper and appreciated the way the authors advance knowledge within the arts & humanities and privilege the perspectives of professional artists on co-creative work.

  

1. Bogart, A. (2021). The Art of Resonance. Methuen Drama.
2. Bourriaud, N. (2002). Relational aesthetics. Presses du réel.
3. Lukić, D. (2023). Dementia as a material for co-creative art making: Towards feminist posthumanist caring. Journal of Aging Studies, 67, 101169. https://doi.org/10.1016/j.jaging.2023.101169
4. Lukić, D., & Mittner, L. (2023). Diffracting Dementia: Co-Creative Experiments with Agential Realism and Multisensoriality in a Residential Care Home in Northern Norway. In E. Just, M. Udén, V. Weetzel, & C. Åsberg (Eds.), Voices from Gender Studies: Negotiating the terms of academic production, epistemology, and the logics and contents of identity. Routledge. https://www.routledge.com/Voices-from-Gender-Studies-Negotiating-the-Terms-of-Academic-Production/Just-Udn-Weetzel-sberg/p/book/9781032415826
5. Lotherington, A. T. (2023). Gender awareness and feminist approaches in dementia studies. In J. R. Fletcher & A. Capstick (Eds.), A Critical History of Dementia Studies. Routledge.
6. Mittner, L. (2021). Resonating moments. Exploring socio-material connectivity through artistic encounters with people living with dementia. https://doi.org/10.1177/14713012211039816
7. Mittner, L., & Gjærum, R. G. (2021). Situating an Art Intervention with People Living with Dementia in a public space. Nordic Journal of Arts, Culture and Health, 3(01–02), 116–121. https://doi.org/10.18261/issn.2535-7913-2021-01-02-11
8. Ward, R., & Sandberg, L. (Eds.). (2023). Critical dementia studies: An introduction. Routledge.
9. Østern, T. P., Jusslin, S., Nødtvedt Knudsen, K., Maapalo, P., & Bjørkøy, I. (2021). A performative paradigm for post-qualitative inquiry. Qualitative Research, 21(3), 14687941211027444. https://doi.org/10.1177/14687941211027444

Is the work clearly and accurately presented and does it cite the current literature?

Yes

If applicable, is the statistical analysis and its interpretation appropriate?

Not applicable

Are all the source data underlying the results available to ensure full reproducibility?

No source data required

Is the study design appropriate and is the work technically sound?

Yes

Are the conclusions drawn adequately supported by the results?

Yes

Are sufficient details of methods and analysis provided to allow replication by others?

Yes

Reviewer Expertise:

Arts-based research, feminist theories, performative research, arts & dementia

I confirm that I have read this submission and believe that I have an appropriate level of expertise to confirm that it is of an acceptable scientific standard.

# Response

**Published:** 2024-04-11

**Authors:** Julian West

**Affiliations:** Open Academy, Royal Academy of Music, London, England, UK

Thank you very much indeed for your insightful review and for your questions – these are much appreciated. Your review has helped us to clarify our thinking in a number of ways, and we have made changes to the article as a result.   We would like to respond to your questions and points in turn. **Can everyone become an artist?** In the ‘With All’ group during a co-creative session there was no strong distinction between the artists and everyone else who participated and to this extent everyone was an artist. However, in this article our specific focus has been on those members of the group who had received training as professional artists and therefore have a more practised and explicit facility with musical and dance skills and knowledge. We have been interested in HOW these skills and this knowledge is deployed within the group. Some sentences have been added to this effect in the section Artists and Art Projects.   You also ask the question, *what would it mean for research design if one person could have different roles during the research process? If roles are not fixed, how can the specific knowledge/skill you are investigating, be captured in the data? *This is a very interesting question, and we have some experience (within other projects) of working with people with dementia who are ALSO professional artists. However, in terms of ‘With All’ (the case being investigated in this paper) there was a distinction between those who were artists (musicians / dancers) and others, and the former are our focus here. We recognise that this is complex in terms of our discussion about the non-hierarchical nature of co-creative arts groups for people with dementia and this is something we start to address in the Discussion. **How does authenticity connect to the master narrative?** Thank you for your interesting thoughts about authenticity. We have clarified this in the text as it refers specifically to the artists’ sense of themselves, their ways of working honestly, openly and with integrity such that this allowed them to have meaningful interactions through their music / dance. We are not thinking about authenticity in relation to the art created but recognise that this was not clear in the first draft. **What is it that people are engaging with?** This was also highlighted by Robyn Dowlen in her review,  and we have clarified the sentence to outline that we mean that arts projects allow people living with dementia to continue engaging in meaningful interactions. This now reads thus:  ‘What has increasingly been recognised is that despite the inability to cure this condition, art projects are one way of enabling people with dementia to continue to participate in social life and have meaningful interactions ( Mittner, L. 2021, Tischler et al, 2023, Zeilig et al, 2019). Moreover, co-creative arts projects have also shown that the arts can offer new and important ways of being in the world with a dementia (Mittner,L, 2021 & Lukić, 2023).’   Thank you for referring us to the work of those in critical dementia studies, we have added references where applicable. **Minor comments** **1. Abstract** Thank you for this observation – we have added the following research question to the abstract: ‘How do professional artists apply their skills and knowledge in co-creative arts groups with people with dementia?’ **2. Introductions and Artists and Art Projects** Thank you for pointing out that we need to include references to the Resonance project and to Artful Dementia Research Lab projects. This was an oversight in the first draft, and these have now been included.   Quotation marks have been amended. **3. Method** The need for more information about the music and dance sessions was also noted by Robyn Dowlen. We have now added further details about the ‘case’ that was central to this paper and brief details about the music and dance session.   With regard to your questions about whether all authors were involved in data collection; the authors were all involved with creating the data (through their journaling and observations) but they were not responsible for collecting this (this was done by the two first authors). Knowledge creation in terms of the focus of this paper did not happen through the co-creative art making rather this took place reflectively, afterwards. **4. Results** We agree that the themes are indeed highly interconnected and have amended Figure 1 to more accurately represent the ways in which they interconnect as a network. **5.Discussion** Concerning your point about the aesthetic framework within which the artists were operating, the musicians were all trained at UK based conservatoires and the dancers in UK and US dance schools. They all work within a Western aesthetic framework, and we have acknowledged this in the Limitations section – this was also mentioned by Robyn Dowlen. We have also added reference to relational aesthetics as far as this is relevant to our work.   We agree that there is the possibility of reinforcing hierarchical distinctions if we privilege professional artists as the sole or only art makers within a co-creative arts project. We have considered this in the discussion and found the use of the concept of making art ‘alongside’ one another not necessarily to the same ends (as noted by Lukić, 2023 in turn quoting Latimer, 2013 and Zeilig et al, 2018) is pertinent for explaining difference in role and recognising complexity rather than privileging the artist over other participants.     We agree with your point about the need to foreground the reciprocity in co-creative practices more clearly. People living with dementia also enabled the artists to take creative risks, and this has been included in the discussion.   You ask the question about the following sentence form the first draft: ‘This sense of togetherness importantly brought coherence to the acts of co-creation and simultaneously was an essential means of facilitating the co-creation.’ On reflection we think this is unclear and detracts from the main point being made concerning collective belonging. We have therefore deleted it. **6. Conclusion** Thank you for your suggestion that this study is better described as an explorative study – we have amended this.
